# Control of Post-War Venereal Disease

**Published:** 1919-01-11

**Authors:** 


### A THREATENED PLAGUE.

### Control of Post-War Venereal Disease.

Probably one of the most discussed topics of the hour is demobilisation and its manifold results. To most it means pleasant anticipation of the resumption of former routines and ambitions with energy and vigour born of new-found experience, the first steps in development of a finer, greater, more virile Britain. But the War has brought more than one baneful influence to bear upon those who have won through its hardships and, among the most insidious, comes the widespread dissemination of venereal disease. Up to the present, the menace has been largely under the control exerted by naval and military authorities, but with the homecoming of our millions and their return to urban and rural life, the greatest of dangers appears to threaten the nation. No more timely warning could be given than the letter recently appearing in the *Times*, and bearing the signatures of some of the most eminent physicians, headed by Sir William Osler. Preparedness is the greatest necessity, delay the most likely pitfall.

During the War there has been ample proof that simple sanitary measures, combined with organised instruction in their application, can work miracles in the control of disease. In the case of venereal infection, the remedies, technique, orders, and prophylactic measures are straightforward enough, and give such a high percentage of efficiency that much of the argument concerning their finer points seems out of place in the present emergency. It is not alone in medical but in national instruction that the secret lies. It has been possible to lecture freely and issue frank printed matter to a large number of men on service, without fear of opposition from those at home who predict an anti-moral effect from such publicity. The methods are now proved and the only regret arises from the fact that exigencies of the fighting have made it impossible to include all the forces concerned. As it is one stands aghast at the probabilities, had these active measures not been taken, for, although a great deal has been effected, many cases from so great a mass must inevitably escape the net and bring fresh, virulent strains back to their native land. This influx will, in spite of all precautions, have been enormously intensified at the completion of demobilisation.

Any M.O., ashore or afloat, will at once describe the difficulty, in spite of its being a punishable offence, of preventing concealment, and here we come to a marked flaw in the home system, where the diseases are not even notifiable in the strict sense of the term. The service way combines the following excellent methods: Periodic examination of all men, isolation of infectious cases with absolute stoppage of leave until active symptoms have passed, treatment at specially equipped centres up to a stage of complete cure when possible, explanatory lectures and pamphlets by medical officers, special attempts to advise the younger men and "boys" and the prohibition of areas known to be particularly dangerous. Yet, taking any one of these, every officer concerned can give numberless instances of breakdown. Even service discipline backing the soundest of schemes has signally failed to make the control "absolute," and without doubt the doctors in this country will very shortly have the result thrust upon them.

Perhaps the greatest danger comes from those once cured, but, owing to general rush of circumstances, relapsed unobserved or those that will do so some months after reaching home. Particularly is this likely in the instance of gonorrhœa, where accepted standards of cure decidedly vary with the personal equation. Have all final examinations been carried out to the full ideal principle that the absence of obvious pain, inflammation and discharge does not necessarily mean that the germ has been exterminated? Then in the case of syphilis: how many men are allowed to depart after injections of mercury, combined or not with sundry doses of intravenous arsenic, producing one negative Wasserman reaction? Only too frequently circumstance does not permit of making of a control test some months later, and it is widely known that a large percentage of these patients return to a positive reaction and a power to transmit the disease, at least by heredity. In either service such happenings may be repeatedly unearthed, and although it is a gross libel to include the large institutions, staffed by specialists, yet it is often the infected's good fortune to reach these places rather than his inevitable fate. To a large extent the War makes its own excuses, and we, realising the impending situation, profiting by experience, should make it our duty to complete a work uncommonly well begun.

To turn, then, to the conditions at home; broadly speaking, the change will comprise a greatly increased case-incidence, probably increased virulence and the appearance of the disease in previously exempt localities. In the first place, this will obviously call for better and wider counter-measures; in the second, for very much more efficient organisation of the whole.

No one would deny to an infected person the advantage of the best that medical science has to offer, for this is shown by the fact that the official plan for making all such benefits easily accessible to the poorest has met with general public approval. But there still remains the well-known opposition to publicity, the hindrance from which the service authorities have been so largely and so mercifully free. Some time ago, Sir Bryan Donkin, in a letter to the *Times*, asked what would happen if syphilis and gonorrhœa were not diseases transmitted by sexual irregularities and had prophylactic measures, including the development of disease after exposure, been devised and proved by crucial experiment to be highly efficacious? There can be no doubt that, in such event, the whole matter would rapidly have become an integral part of the health education of the public. At the moment, however, we are far below that ideal. All are agreed that people should become enlightened as to the dangers and sequelæ of infection, but still prophylasis must remain unmentionable.

Up to the present, the approved instruction has taken the form of true and rightly terrifying descriptions of the complications and results of contracting the disease, a method logically bound to do a great deal of good since the public concerned must assuredly take the whole subject far more seriously than at quite recent dates, when perfectly amazing indifference and ignorance were too often shown. But still it is tackling a purely scientific problem from a moral standpoint and practically attempting to aid the person's natural conscience and will-power by a wholesome fear of the consequences of a lapse from the path of virtue. The means of extending any such teaching, whatever its degree, are open to much debate,

**A THREATENED PLAGUE**—*(continued)*

but in the first place, as urged by the Royal Commission, every medical student—and all members of any scientific profession remain students to the end—should obtain adequate, up-to-date knowledge of all aspects of venereal disease, for it is from the doctor that any really efficient propaganda must emanate and the action of the British Medical Association and Council in securing legislation making it a penal offence for any person other than a qualified practitioner to treat these ailments has placed the power in the right hands and removed one of the greatest aids to concealment. The problem before the profession is first to control the disease, then to eradicate it from the country as far as possible. Success has been considerable, both here and abroad, with smallpox, typhoid and many of the most fatal infections known, and what has been the key to these victories?—not the people's fear of the consequences. Certainly general spread of knowledge in the elements of hygiene has contributed, but that applies equally to almost any microbic invasion. The secret lies not only in knowing exactly where and when the cases arise, *i.e.*, in notification, but also, absolutely paramount, in prophylaxis.

Against the former is raised a positive host of objections, but all of them seem to have a purely social origin, while the latter is considered likely to be so anti-moral in its effects that the Committee of the Medical Council decided it was "undesirable to take any steps in favour of prophylactic teaching." Of course, during the last two and a half years, great strides have been made in increasing the number of treatment centres and clinics under Local Government Board supervision, but even if it were true that with these we had obtained a good check on the incidence, there is fast approaching a time when their number will be just as relatively deficient as before. Left to work under existing restrictions we are at an impasse, for it means fighting an oncoming tide with only half the possible measures at our disposal.

In pleading for freedom of action it may be granted that the necessary information really is becoming very widely diffused indeed, but at the same time it must be noted that, judging by the lay press of the last few years, there is not the least hesitation in publishing, where the youngest may read it, matter which would formerly have come perilously near to provoking the censor's pencil and that nowadays spiritual teaching is guided by greater freedom of thought than ever before. Could we not assume that the public have gained such "knowledge of the world" and knowledge of themselves as warrants the taking of one step further—at least for those old enough to instruct the younger generation? It is admitted that access to prophylactic means may in a number of cases lead to irregularity of conduct when the person is presented with the possibility of avoiding the risk of his actions; but surely when these excellent new clinics are seen full to overflowing, the realisation comes abruptly home that, had the majority of the people had even the most abridged instruction in the means at their disposal much of the infection would never have occurred.

The price is a certain number tempted to misconduct; but is it quite certain that this number is so great as to outweigh the saving of hereditary disease, the diminished birth-rate as foretold by these crowded out-patients, and the immediate limitation of contact spread which must result from preventative methods? General teaching is here said to work in a vicious circle, but, by partially withholding it, we seem to allow the infection to run in such a course as gives the maximum of adult cases and so of inborn disease. Consider only the health of the nation, carry all possible counter-measures to an efficient extent, and it might reasonably be hoped to reverse such a tendency.

But let us return to notification and what in another disease we should call contacts. Venereal taint, being a social disgrace, brings its notification to mean a certificate of the same. The public may object, but do they—or the authorities—retain their faith in the extent of the profession's tact and discretion? There does not seem to be any reason, given a good distribution of clinics, each under the control in both out-patient and laboratory departments, why the minimum of publicity should not obtain since any patient may drop his name for a number at the entrance of a hospital and divulge the identity of the two only when in private with his doctor. The investigating centres would accept cases, as they do now, from a distance and be preferably attached to general institutions. In this way no one is unnecessarily obvious to his neighbours at home and, the diagnosis once established, can be referred privately to a "Venereal Officer" of his district, who, if he does not happen to be in regular attendance on the person, can secure much family and contact examination by tactful investigation on the part of the practitioner concerned. No scheme could ever give absolute secrecy, but by some such methods, aided by a penalty for wilful concealment, would give as much privacy as is now accorded to many equally noxious conditions and yet provide the medical man with that exact information of incidence essential to any organised work. As things stand to-day one must admit that there is some justice in the criticisms regarding the obviousness of some centres, but really very slight imagination and effort could speedily bring about the refinements needed, and that accomplished, opposition might weaken enough to allow the use of some direct methods as employed in all other infectious disease.

The question of prostitution and medical examination must be passed over, but it is well to recall that other nations have practical ways of dealing with this part of the menace—why has not Great Britain?

In conclusion, we may briefly return to the contrast with the service plans. They have the enormous advantage of being, if not quite absolute, very nearly so and their relatively better control is their reward. At home the finest of medical minds urge us to activity. Is it not time that a clean sweep was made of all these obstructions and the full force of our means utilised? Given freedom of action, an immensity of good might be accomplished, for though the work to be done is stupendous, it should not be impossible of achievement. The public are soon to suffer yet another onslaught by disease and they may well be made partners in the scheme for their salvation. Surely the danger and the opportunity have arrived together.

